# Melioidosis presenting with mediastinal lymphadenopathy masquerading as malignancy: a case report

**DOI:** 10.1186/1752-1947-6-28

**Published:** 2012-01-23

**Authors:** Kavitha Saravu, Chiranjay Mukhopadhyay, Vandana Kalwaje Eshwara, Barkur Ananthakrishna Shastry, Kundapura Ramamoorthy, Sushma Krishna, Vishwanath Sathyanarayanan

**Affiliations:** 1 Department of Internal Medicine, Kasturba Medical College, Manipal University, Karnataka, India; 2 Department of Microbiology, Kasturba Medical College, Manipal University, Karnataka, India; 3 Department of Microbiology, Amrita Institute of Medical Sciences, Cochin. Kerala, India

**Keywords:** melioidosis, mediastinal mass, lymphadenopathy, malignancy, tuberculosis

## Abstract

**Introduction:**

Melioidosis, endemic in Thailand and in the Northern Territory of Australia is an emerging infectious disease in India which can present with varied forms. A case of melioidosis, presenting as a rare anterior mediastinal mass which can masquerade as malignancy or tuberculosis, is described here. With treatment, our patient initially showed an increase in the size of mediastinal node and development of new submandibular node.. To the best of our knowledge, this phenomenon has not been documented in the literature and the same is highlighted in this case report.

**Case Presentation:**

A 43-year-old Asian man with diabetes presented with fever, loss of appetite, weight loss for one month and painful swelling below his left mandible for five days. An examination revealed an enlarged left submandibular lymph node and bilateral axillary lymph nodes. A chest X-ray showed mediastinal widening. Computed tomography of his thorax showed a lobulated heterogeneously enhancing anterior mediastinal mass encasing the superior vena cava suggestive of malignancy. An excision biopsy of the lymph node showed granulomas suggestive of tuberculosis but bone marrow culture and lymph node aspirate culture grew *Burkholderia pseudomallei*. He was treated with parenteral ceftazidime and amoxicillin-clavulanic acid. During the course of treatment, he developed an enlargement of the submandibular lymph node on the opposite side. It gradually subsided with the continuation of therapy orally with a combination of cotrimoxazole and doxycycline for six months. A repeat computed tomography chest scan showed resolution of the mediastinal mass.

**Conclusion:**

Melioidosis can present as a mediastinal mass that mimics tuberculosis or malignancy. During the initial phase of treatment of melioidosis, the appearance of new lymph nodes or an increase in the size of the existing lymph nodes does not mean treatment failure. Inexperienced clinicians may consider this as treatment failure and may switch treatment. To the best of our knowledge, this is the first report documenting this phenomenon in melioidosis cases.

## Introduction

Melioidosis is called a mimicker of maladies. In its acute form it can mimic any community acquired bacterial sepsis, pneumonia or abscess, especially that produced by staphylococcus. In its chronic form, it can mimic tuberculosis or malignancy [1]. Melioidosis can present with subcutaneous abscesses and visceral abscesses in the liver, spleen, prostate, parotid, and lymph nodal mass [2,3].

Melioidosis is endemic in Northern Australia, Thailand, Singapore, Malaysia, Myanmar and Vietnam [3]. In India, it is sporadic with an increasing trend in the southern states [4]. *Burkholderia pseudomallei*, the causative organism, is a gram negative, motile bacillus isolated from soil and surface water. The disease is acquired by inoculation through abraded skin, inhalation or ingestion [5]. The majority of cases present during the rainy season [6]. The incubation period ranges from 24 hours to many years [3]. In an Australian study, chronic renal disease, chronic lung disease, and age > 45 years were independent risk factors for melioidosis [7]. It produces necrotizing inflammation, abscess or granuloma with multinucleated giant cells [8]. Clinical disease may present acutely with fever of less than two months duration or chronically with more than two months of fever with or without other symptoms such as cough, discharging sinus, and subcutaneous swellings. Localized disease presents as skin ulcers and subcutaneous abscesses or pneumonia. Disseminated disease can present with pneumonia, abscesses in the liver, spleen, kidney, prostate, skin and subcutaneous tissue, septic arthritis and osteomyelitis with or without septicemia. Lymph node swelling with necrosis can occur as part of either disseminated melioidosis or its local forms [3,4]. However, mediastinal lymphadenopathy as a presenting feature of melioidosis is rare. We present a case of a patient with melioidosis which involved the mediastinal lymph node and mimicked malignancy and tuberculosis.

## Case presentation

**Figure 1:**
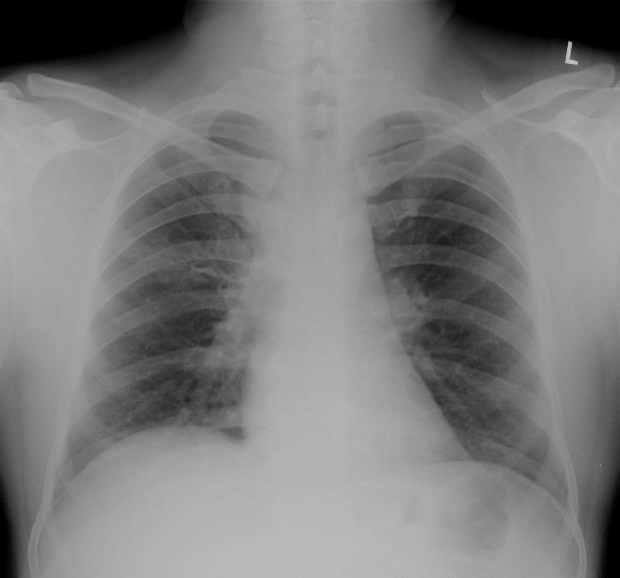
**Chest X-ray showing widening of the right paratracheal stripe**.

**Figure 2:**
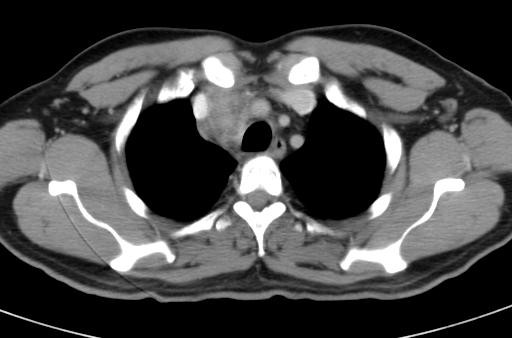
**Computed tomography chest scan showing anterior mediastinal mass in the right paratracheal region**.

A 43-year-old Asian man with diabetes, a clerk in an office, presented with fever of one month and painful swelling below the left submandibular region of five days duration with a history of weight loss and poor appetite. An examination revealed a febrile patient with multiple lymph node swellings. His left submandibular lymph node was 2 × 1 cm and tender. His bilateral axillary nodes were palpable, the largest being 0.5 × 0.5 cm. Vital signs and systemic examination were normal. Investigations revealed neutrophilic leukocytosis with high erythrocyte sedimentation rate and uncontrolled diabetes mellitus. His chest X-ray showed widening of the right paratracheal region probably due to lymph node enlargement (Figure 1). He was empirically started on intravenous amoxicillin-clavulanic acid and subcutaneous insulin. A computed tomography (CT) scan of his thorax showed an anterior mediastinal mass measuring 4.2 × 3.7 × 3 cm in the right paratracheal region suggestive of a malignant mass with a few upper lobe densities (Figure 2). An excision biopsy of the submandibular swelling showed a non-caseating granuloma composed of lymphocytes and plasma cells suggestive of tuberculosis. A tuberculin skin test was negative. His bone marrow did not show acid fast bacilli or granuloma. Anti-tuberculosis treatment was not administered. Blood culture and bone marrow culture grew *B. pseudomallei *sensitive to amoxicillin-clavulanic acid, ceftazidime, piperacillin, cefaperazone-sulbactum, co-trimoxazole, doxycycline, tetracycline, meropenem and imipenem. He was treated for two weeks with ceftazidime (2 gm every six hours intravenously). During his hospital stay, he developed new right submandibular lymhadenopathy. However, the same treatment was continued. He became afebrile in eight days. He was discharged with oral co-trimoxazole (40/8 mg/kg) and doxycycline (100 mg) every 12 hours. At one month, a CT scan of his thorax revealed an increase in the right paratracheal lesion with the resolution of upper lobe densities. Treatment was continued with oral co-trimoxazole and doxycycline for six months and a repeated computed tomography (CT) scan of his chest was normal.

## Discussion

Melioidosis cases can present as a mediastinal mass mimicking malignancy or tuberculosis. This is because of enlarged mediastinal lymph nodes with necrosis. They must be evaluated by fine needle aspiration and cytology (FNAC) or biopsy. Mediastinal lymphadenopathy has been reported in 3% of patients in a 20-year Darwin prospective study [9]. Few other cases are reported in the literature with mediastinal lymphadenopathy [2,10]. This could be secondary to inhalation or due to hematogenous spread following inoculation.

Chronic melioidosis can be confused with tuberculosis clinically as well as histopathologically. Histopathology of these lesions can show granulomas. In our case it was a non-caseating granuloma and the tuberculin skin test was negative. The growth of *B. pseudomallei *settled the diagnosis in our patient and hence the ability to identify the growth is of paramount importance. This case report highlights the fact that encasement of the superior vena cava can occur occasionally in non-malignant conditions.

The initial intensive phase of parenteral therapy for melioidosis is with ceftazidime 50 mg/kg (up to 2 g) every six hours or meropenem 25 mg/kg (up to1 g) every eight hours, or imipenem 25 mg/kg (up to 1 g) every six hours with or without sulfamethoxazole/trimethoprim 40/8 mg/kg (up to 1600/320 mg) every 12 hours for 10 to 14 days. Deep seated abscesses extensive pulmonary disease, osteomyelitis, septic arthritis and neurological melioidosis require four to eight weeks of the intensive phase. Subsequent oral eradication therapy is required to prevent recrudescence or relapse. Sulfamethoxazole/trimethoprim 40/8 mg/kg (up to 1600/320 mg) every 12 hours for three to six months is recommended [3].

Another interesting feature of our case is the appearance of new submandibular swelling while our patient was receiving appropriate therapy. There was an increase in the size of mediastinal lymphadenopathy as evidenced by a repeated CT scan of thorax done at one-month follow up. In a review by White, the author opined that the enlargement of an abscess or appearance of new abscesses, especially in skeletal muscle, or seeding to a joint, is not uncommon in the first week of treatment, and is not necessarily a sign of treatment failure [5]. However, we did not find any documentation of this phenomenon in the literature. Two mechanisms can be put forth for this. The first is the initial progression of the infection which may sometimes be seen despite the use of appropriate antibiotics and its eventual resolution. Another concomitant or additional mechanism is that lymph node enlargement may represent an immune mediated response to infection, something similar to the paradoxical reaction seen in tuberculosis.

## Conclusion

The appearance of new lymph nodes or an increase in the size of the existing lymph nodes does not mean treatment failure and the appropriate treatment has to be continued. Melioidosis can mimic tuberculosis and malignancy and always has to be considered in the differential diagnosis of lymphadenopathy with or without fever in endemic areas or in travellers returning from endemic areas.

## Consent

Written informed consent was obtained from the patient for publication of this case report and any accompanying images. A copy of the written consent is available for review by the Editor-in-Chief of this journal

## Competing interests

The authors declare that they have no competing interests.

## Authors' contributions

KS, BAS and VS managed the patient. KS, CM, VKE and RK prepared and edited the manuscript. SK and RK helped in data collection. All authors read and approved the final manuscript
